# Upper gastrointestinal tract involvement is more prevalent in Korean patients with pediatric Crohn’s disease than in European patients

**DOI:** 10.1038/s41598-020-75938-1

**Published:** 2020-11-04

**Authors:** Eun Sil Kim, Yiyoung Kwon, Yon Ho Choe, Mi Jin Kim

**Affiliations:** Department of Pediatrics, Samsung Medical Center, Sungkyunkwan University School of Medicine, 81 Irwon-ro, Gangnam-gu, Seoul, 06351 Korea

**Keywords:** Gastroenterology, Medical research

## Abstract

In pediatric Crohn’s disease (CD) patients, it is important to define the disease phenotype at diagnosis for stratifying risk. In this retrospective study, we aimed to assess the disease phenotype compared to EUROKIDS registry and analyze disease outcome of pediatric CD patients according to upper gastrointestinal (GI) tract involvement. A total of 312 patients were included. The median age at diagnosis was 13.7 years and 232 patients (74.4%) were identified to have upper GI involvement at diagnosis. In Korean pediatric CD patients, there were significant differences in male predominance (72.8% vs. 59.2, *p* < 0.001), proportion of upper GI involvement (74.4% vs. 46.2%, *p* < 0.001), and perianal disease (62.1% vs. 8.2%, *p* < 0.001) compared to data in the EUROKIDS registry. Younger age (OR 2.594, *p* = 0.0139) and ileal involvement (OR 2.293, *p* = 0.0176) at diagnosis were associated with upper GI involvement. There were no significant differences in disease outcomes between patients with and without upper GI tract involvement. This study revealed that upper GI involvement is more prevalent in Korean patients with pediatric Crohn’s disease than in European patients, and the disease outcome did not appear to differ according to upper GI tract involvement.

## Introduction

Crohn’s disease (CD) is a multifactorial idiopathic chronic disease of the gastrointestinal (GI) tract with complex pathophysiology including genomes and immune responses, and variable clinical manifestations and disease course^1,2^. Approximately 25% of the patients with CD are diagnosed during childhood and adolescence. The incidence in pediatric patients has increased significantly over the past decades and the age at diagnosis of CD is also getting younger^3–6^.

To select the optimal treatment and assess the prognosis of pediatric CD patients, it is important to clarify the factors influencing poor outcomes. It is known that deep colonic ulcers, extensive disease, growth retardation, B2 and/or B3 disease behavior at the onset, severe osteoporosis, and severe perianal disease are potentially predictive of poor outcomes^7^. In addition, the GETAID cohort study identified young age, upper GI tract and rectal involvement (but not colonic or ileal), and B2 and/or B3 disease behavior as prognostic factors for poor outcomes over 15 years of disease^8^. Therefore, in pediatric CD patients, it is important to define the disease phenotype at diagnosis for stratifying risk and prognostic factors.

In Korea, there are scarce data on the disease phenotype of the entire GI tract and the disease course according to disease phenotype in pediatric CD patients^9,10^. The primary aim of the present study was to assess the disease phenotype of pediatric CD patients and investigate the characteristics including upper GI tract involvement. The secondary aim of this study was to find the factors associated with upper GI tract involvement in pediatric CD patients and differences in the disease course according to upper GI tract involvement.

## Methods

### Patients and study design

This study was a retrospective, observational cohort study conducted at the Department of Pediatrics of Samsung Medical Center between January 2004 and July 2019. The subjects were pediatric CD patients diagnosed at < 19 years of age with available data and evaluation of the entire GI tract. CD was diagnosed in accordance with the revised Porto criteria of the European Society for Pediatric Gastroenterology, Hepatology, and Nutrition, which implies that small bowel evaluation, as well as esophagogastroduodenoscopy (EGD) and ileocolonoscopy, were performed in all subjects^11^. The entire GI tract evaluation consisted of EGD, ileocolonoscopy, and appropriate imaging of the small bowel including capsule endoscopy, and/or magnetic resonance enterography (MRE). Also, there were complete follow-up data for at least one year after diagnosis in all subjects. Patients diagnosed with ulcerative colitis, inflammatory bowel disease (IBD)-unspecified, diagnosed age at ≥ 19 years old, and those with missing baseline clinicodemographic data were excluded. Disease classification and behavior were based on the Paris classification^12^. The inclusion criteria for this study were the same as those for the EUROKIDS study to enable comparison of the disease phenotypes^13^.

Demographic information and the clinical data at diagnosis, including sex, diagnosis age, age at symptom onset, duration of disease, disease phenotype including the entire GI tract, previous history of perianal or CD-related GI tract surgery before diagnosis, and a first-degree family history of IBD, were collected from the electronic medical records. The severity of the disease was evaluated through the Pediatric Crohn’s Disease Activity Index (PCDAI)^14^, laboratory results, and the simple endoscopic score for CD (SES-CD).

To evaluate disease outcomes according to upper GI tract involvement and small bowel (jejunal) involvement, the need for CD-related intestinal surgery and perianal fistula/abscess surgery was assessed.

### Definitions

**Figure 1:**
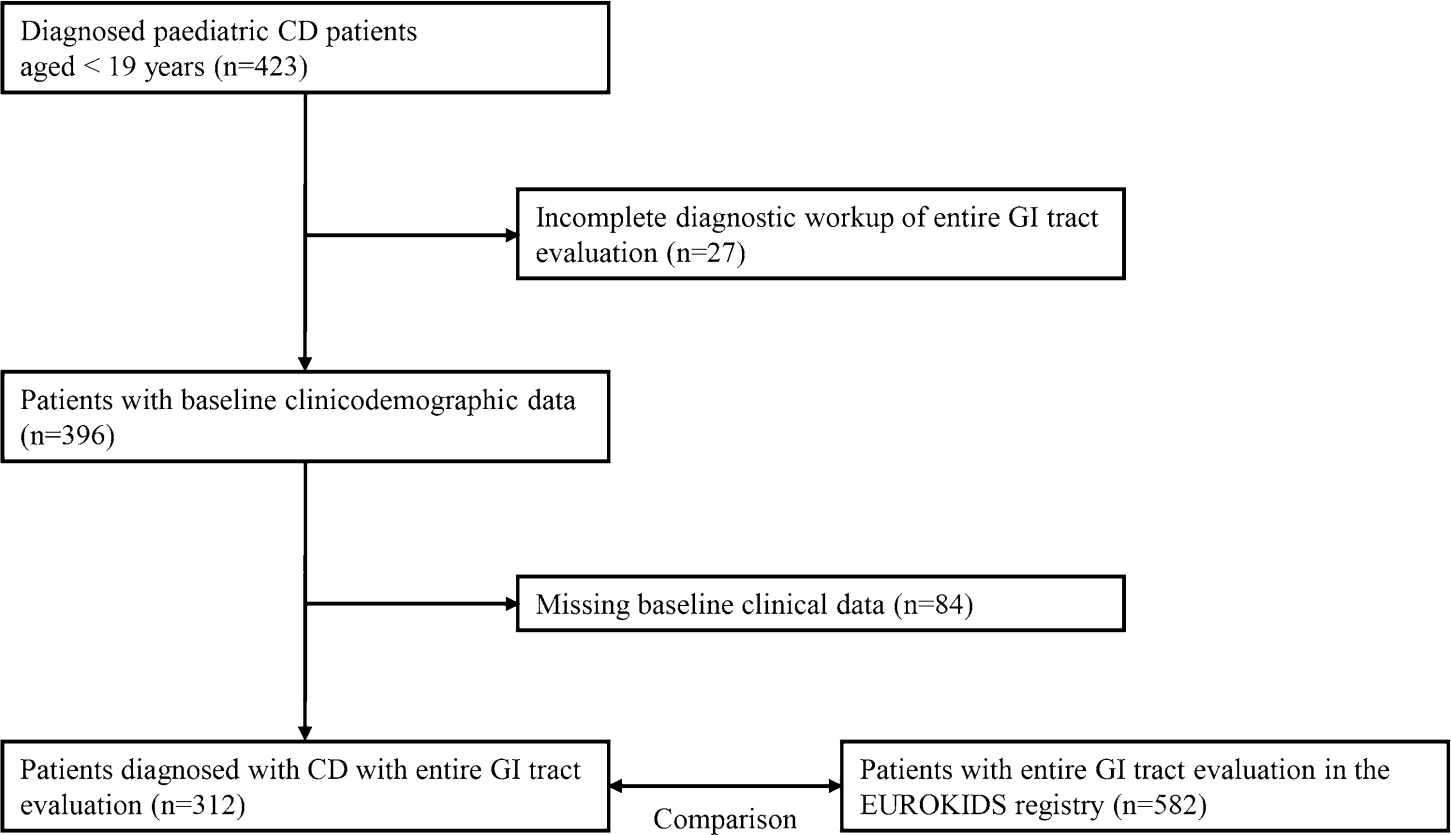
Study flow diagram.

The diagnosis of CD was made by physicians in a single center based on clinical symptoms, laboratory results, endoscopic appearance, histologic findings, and small bowel imaging studies. The disease phenotype at diagnosis was categorized according to the Paris classification^12^ and compared with the EUROKIDS registry^13^ (Fig. 1).

The location of GI tract involvement in CD was determined by the presence of ulcers, erosions, cobblestoning, and/or luminal narrowing^15–17^. The GI tract from the esophagus to the second portion of the duodenum was evaluated by EGD, and from the terminal ileum to the rectum by ileocolonoscopy. In the remaining small bowel, disease involvement was determined by findings on small bowel imaging and/or capsule endoscopy. Upper GI tract involvement (L4) was defined as disease from the esophagus to the proximal 2/3 of the ileum and lower GI tract involvement was defined from the distal 1/3 of the ileum to the rectum according to the Paris classification system^12^. Upper GI disease was further divided into esophagoduodenal (L4a), jejunal/proximal ileal (L4b), or both (L4ab). We regarded nonspecific macroscopic findings, such as erythema, edema, and/or nodularity in the esophagogastroduodenum (L4a), as insufficient evidence of upper GI tract involvement. Endoscopic findings on the terminal ileum were considered more accurate than small bowel studies in the case of disagreement. Perianal disease was defined as a perianal abscess and/or fistula, and isolated skin tags, fissures, or hemorrhoids were excluded.

### Statistical analysis

Continuous variables are presented as medians and the interquartile range (IQR) for non-normally distributed data and categorical variables are presented as number and proportion. For statistical comparison between the groups according to upper GI involvement and between Korean cohort and ERUOKIDS registry, the t-test or Wilcoxon rank-sum test was used for continuous variables and the chi-squared test or Fischer’s exact test was used for categorical variables. Univariate and multivariate logistic regression analyses were conducted to examine the association between upper GI involvement and the variables. Factors with *p* < 0.1 in the univariate analysis were included in the multivariate analysis using a stepwise selection procedure. The results are expressed as adjusted ORs with 95% confidence intervals (CIs). Kaplan–Meier survival analysis was used to evaluated surgery free survival stratified by upper GI tract involvement. All statistical analyses were carried out using Rex (Version 3.0.3, RexSoft Inc., Seoul, Korea).

### Ethics declarations

This study was approved by the Institutional Review Board of the Samsung Medical Center (IRB file No. 2020-01-033). All patients and A parent and/or legal guardian of subjects who are under 18 provided written informed consent. We confirmed that all methods were performed in accordance with the approved guidelines and regulations. We reported and presented data according to the STROBE statement.

## Results

### Baseline characteristics of patients with and without upper gastrointestinal involvement

From January 2004 and July 2019, a total of 312 pediatric patients were diagnosed with CD in accordance with the revised Porto criteria and were eligible for analysis. The median age of the patients was 13.7 years (IQR 11.9–15.7, 89.7% was ≤ 16 years old), with a median diagnostic delay (time interval from the onset of the first symptoms to the establishment of a CD diagnosis) of 5.4 months (IQR 2.4–12.8). Of the patients, 227 (72.8%) were male and the median follow-up duration was 6.6 years (IQR 4.1–9.9 years). Eighteen patients (5.6%) had previous bowel resection history and 102 patients (32.7%) had previous anal fistula or perianal abscess surgery before diagnosis. Twenty patients (6.4%) had first-degree relatives with IBD. The other baseline characteristics are summarized in Table 1.

**Table 1:** Baseline characteristics and disease phenotype of pediatric Crohn’s disease patients.

	Total	No Upper GI involvement	Upper GI involvement	*p* value
Observational duration	6.6 (4.1, 9.9)			
Number of patients (%)	312 (100)	80 (25.6)	232 (74.4)	
**Sex (%)**				
Male	227 (72.8)	56 (70)	171 (73.7)	0.6195
Female	85 (2.0)	24 (30)	61 (26.3)	
Age at diagnosis (years)	13.7 (11.9, 15.7)	14.8 (12.2, 16.5)	13.4 (11.6, 15.4)	0.0133
**Age at diagnosis**				
16 or less	280 (89.7)	66 (82.5)	214 (92.2)	0.0236
More than 16	32 (10.3)	14 (17.5)	18 (7.8)	
Diagnostic delay (months)	5.4 (2.4, 12.8)	5.1 (2.5, 12.3)	5.3 (2.4, 12.5)	0.5094
Body mass index (kg/m^2^)	18.3 (16.2, 20.3)	18.4 (16.1, 19.6)	18.2 (16.3, 20.4)	0.4649
PCDAI	32.5 (21.9, 40)	32.5 (22.5, 40)	32.5 (20, 40)	0.8072
SES-CD	14 (7, 22)	14.5 (7, 23.5)	14.5 (7.75, 21)	0.9186
White blood cell count (/uL)	8585 (6800, 10,730)	8325 (6960, 10,730)	8605 (6732.5, 10,667.5)	0.8749
Hemoglobin (g/dL)	12.2 (10.9, 13.2)	12.3 (10.9, 13.2)	12.1 (10.97, 13.2)	0.9656
Platelet count (/uL)	368.5 (298.75, 474.25)	378.5 (304.75, 474.25)	366.5 (297.5, 471.75)	0.699
Erythrocyte sedimentation rate (mm/hr)	45 (20, 71)	44 (19.5, 72.75)	45 (21, 70.25)	0.8914
C-reactive protein (mg/dL)	1.54 (0.39, 4.2)	1.2 (0.36, 3.81)	1.75 (0.41, 4.25)	0.2218
**Lower GI (%)**^a^				
No other location	1 (0.32)	–	1 (0.43)	0.0815
L1	36 (11.5)	8 (10)	28 (12.1)	
L2	41 (13.1)	17 (21.3)	24 (10.3)	
L3	234 (74.8)	55 (68.8)	179 (77.2)	
**Behavior (%)**^a^				
B1	255 (81.2)	70 (87.5)	185 (79.7)	0.5302
B2	37 (11.8)	6 (7.5)	31 (13.4)	
B3/B2B3	20 (6.4)	4 (5.0)	16 (6.9)	
Perianal symptom (%)	170 (54.5)	40 (50)	130 (56.0)	0.4211
Perianal lesion on MRE (%)	193 (62.1)	49 (61.3)	144 (62.3)	0.9688
**Surgical history (%)**				
Fistula/abscess surgery	102 (32.7)	24 (30)	78 (33.6)	0.6476
Intestinal resection	18 (5.6)	5 (6.3)	13 (5.6)	0.786
Family history of IBD (%)	20 (6.4)	2 (2.5)	18 (7.8)	0.1642
Anti-TNF agents	280 (89.7)	67 (83.8)	213 (91.8)	0.0664

*PCDAI* Paediatric Crohn’s disease activity index, *SES-CD* simple endoscopic score for Crohn’s disease, *Anti-TNF agents* Anti-tumor necrosis factor agents.

^a^According to the Paris classificsation.

Among the patients, 232 patients (74.4%) had upper GI involvement at diagnosis. A comparison of the variables between the patients with and without upper GI involvement revealed only a significant difference in younger diagnosis age (13.4 vs. 14.8, *p* = 0.0133) with a higher proportion of A1a/A1b (92.2% vs. 82.5%, *p* = 0.0236). There were no significant differences in other disease phenotypes, disease behavior, disease severity, family history of IBD, surgical history, and anti-TNFα therapy during the study period, as described in Table 1.

### Comparison of disease phenotype with the EUROKIDS registry

Compared to the EUROKIDS registry, there were statistically significant differences in male predominance (72.8% vs. 59.2, *p* < 0.001), first-degree family history of IBD (6.4% vs. 10.8%, *p* = 0.003129), ileal involvement (L1/L3, 86.3% vs. 69.1, *p* < 0.001), upper GI involvement (74.4% vs. 46.2%, *p* < 0.001), and perianal disease (62.1% vs. 8.2%, *p* < 0.001) in this study. The disease behavior according to the Paris classification was comparable between the two studies (*p* > 0.05) (Table 2). Regarding upper GI tract involvement, there were no remarkable differences in the upper GI macroscopic and microscopic findings between the two studies (Table 3).

**Table 2:** Comparison of pediatric Crohn’s disease phenotype at diagnosis between Korean cohort and the EUROKIDS registry.

	Korea	EUROKIDS	*p* value
	n = 312	n = 1221	
**Sex (%)**			< 0.001
Male	227 (72.8)	723 (59.2)	
Female	85 (27.2)	498 (40.8)	
**Ethnicity (%)**			< 0.001
Asia	312 (100)	50 (4.1)	
Caucasian	0	1049 (86.7)	
Arabian	0	43 (3.6)	
African-Caribbean	0	18 (1.5)	
Others	0	50 (4.1)	
**1st-degree family history of IBD**	20 (6.4)	129 (10.8)	0.003129

	n = 312	n = 582	*p* value
**Age at diagnosis (%)**^a^			< 0.001
A1a	24 (7.7)	114 (19.6)	
A1b/A2	288 (92.3)	468 (80.4)	
**Lower gastrointestinal tract location (%)**^a^			< 0.001
No other location	1 (0.6)	21 (3.6)	
L1	36 (11.5)	95 (16.3)	
L2	41 (13.1)	159 (27.3)	
L3	234 (74.8)	307 (52.8)	
**Upper gastrointestinal tract location (%)**^a^			< 0.001
No other location	80 (25.6)	313 (53.8)	
L4a	82 (26.3)	129 (22.1)	
L4b	76 (24.4)	97 (16.7)	
L4ab	74 (23.7)	43 (7.4)	
**Behavior (%)**^a^			0.434286
B1	255 (81.7)	477 (82)	
B2	37 (11.9)	77 (13.2)	
B3/B2B3	20 (6.4)	28 (4.8)	
**Perianal disease (%)**^a^	193 (62.1)	48 (8.2)	< 0.001

^a^According to the Paris classification.

**Table 3:** Comparison of macroscopic and microscopic findings in the upper gastrointestinal tract of pediatric Crohn’s disease patients between Korean cohort and the EUROKIDS registry.

	Korea	EUROKIDS	*p* value
**Number of patients**	**312**	**582**	
**Upper gastrointestinal macroscopic findings (%)**			0.546652
**Esophageal involvement**	14 (4.5)	34 (5.8)	
Ulceration	4	12	
Erosion	11	23	
**Gastric involvement**	62 (19.9)	102 (17.5)	
Ulceration	26	36	
Erosion	45	71	
Cobble stone	0	1	
**Duodenal involvement**	48 (15.4)	100 (17.2)	
Ulceration	18	31	
Erosion	41	72	
Cobble stone	0	2	
Luminal narrowing	2	2	
**Number of patients**	**312**	**427**	
**Upper gastrointestinal microscopic findings-granuloma (%)**			0.121849
Esophagus	3 (1.0)	20 (4.7)	
Stomach	24 (7.7)	49 (11.5)	
Duodenum	2 (0.6)	14 (3.3)	

### Factors associated with upper gastrointestinal tract involvement at diagnosis

According to the univariate logistic regression analysis, the age at diagnosis and the initial location in the lower GI tract were associated with upper GI tract involvement. These factors as well as sex were included in the multivariate logistic regression analysis by stepwise selection and revealed that the age at diagnosis (16 years or less vs. more than 16, OR 2.594, 95% CI 1.214–5.541, *p* = 0.0139) and the initial location in the lower GI tract (ileal vs. non-ileal involvement, OR 2.293, 95% CI 1.156–4.441, *p* = 0.0176) were positively associated with the presence of upper GI tract involvement in pediatric CD patients (Table 4). In addition, during the study period, anti-TNFα therapy was associated with upper GI tract involvement. (OR = 2.292, *p* = 0.0365).

**Table 4:** Univariate and multivariate logistic regression model for the association of upper gastrointestinal tract involvement at the time of Crohn’s disease diagnosis.

Upper GI involvement at diagnosis	Univariate		Multivariate	
	OR [95% CI]	*p* value	OR [95% CI]	*p* value
Sex [female vs. male]	0.711 [0.383–1.320]	0.2795		
Age at diagnosis [16 years or less vs. more than 16]	2.522 [1.190–5.344]	0.0158	2.594 [1.214–5.541]	0.0139
Initial location [ileal vs. non-ileal involvement]	2.234 [1.135–4.400]	0.0201	2.293 [1.156–4.441]	0.0176
Behavior [complicated vs. non-complicated]	1.778 [0.852–3.712]	0.1252		
1st-degree family history of IBD	3.280 [0.744–14.462]	0.1165		
Previous intestinal surgery	0.890 [0.307–2.581]	0.831		
Previous fistula/abscess surgery	1.182 [0.682–2.049]	0.5519		
Diagnostic delay	0.992 [0.982–1.002]	0.1165		
Body mass index	1.057 [0.981–1.140]	0.1441		
Paediatric Crohn’s disease activity index	0.997 [0.979–1.015]	0.441		
Simple endoscopic score for Crohn’s disease	0.994 [0.9678–1.021]	0.6733		
White blood cell count	1.000 [0.999–1.000]	0.6186		
Haemoglobin	1.021 [0.892–1.170]	0.7595		
Platelet count	1.000 [	0.9916		
Erythrocyte sedimentation rate	1.000	0.9751		
C-reactive protein	1.066	0.1282		
Anti-tumor necrosis factor agents therapy	2.175 [1.020–4.637]	0.0442	2.292 [1.053–4.988]	0.0365

### Impact of upper gastrointestinal tract involvement on CD-related complications in pediatric patients

To investigate the impact of upper GI tract involvement on disease course, a follow-up analysis of all patients with upper GI tract involvement and especially jejunal involvement at the time of CD diagnosis was conducted and compared to patients without such involvement. During a median follow-up of 6.6 years (IQR 4.1–9.9), we identified 51 cases (16.3%) with the occurrence of severe complications, in which 27 cases of perianal fistula required fistulectomy and 24 cases of intestinal stenosis or perforation required intestinal resection surgery.

**Figure 2:**
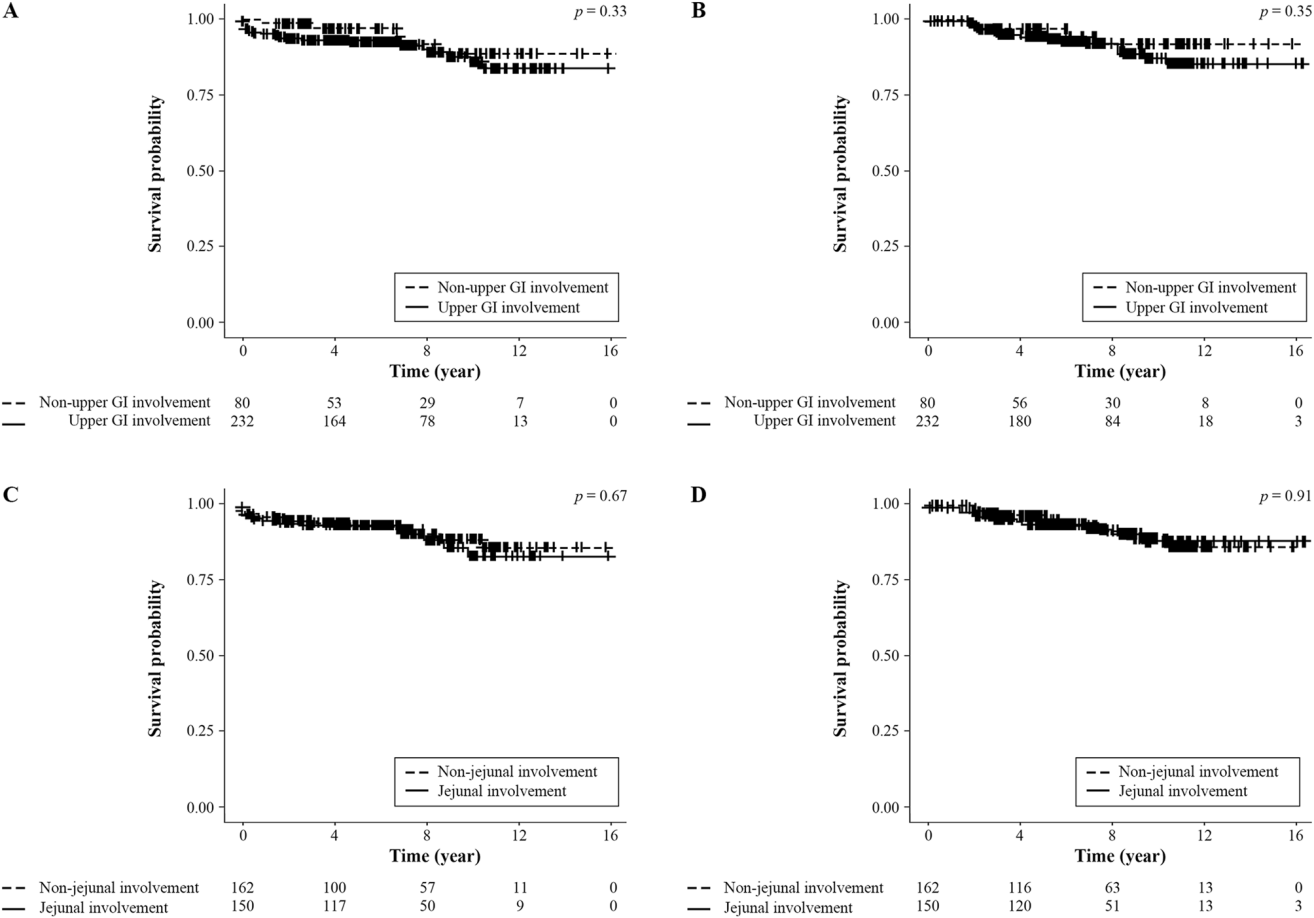
Impact of upper gastrointestinal tract and jejunal involvement on CD-related complications in pediatric CD patients. Kaplan–Meier analysis for perianal surgery (**A**) and intestinal resection (**B**) according to upper gastroinetstinal involvement. Kaplan–Meier analysis for perianal surgery (**C**) and intestinal resection (**D**) according to jejunal involvement.

Forty-two cases were detected in the group of patients with upper GI tract involvement at the time of CD diagnosis (82.4%), and nine cases were observed in the subjects without upper GI tract involvement at diagnosis (17.6%). According to the Kaplan–Meier analysis for complication-free survival, there were no significant differences in severe complications requiring surgery such as fistulectomy or intestinal resection between the patients with upper GI tract involvement and the patients without such involvement (Fig. 2A,B), *p* > 0.05). In addition, the subgroup analysis of patients with and without jejunal involvement revealed no statistically significant differences between the groups (Fig. 2C,D), *p* > 0.05).

## Discussion

To date, a few studies analysing disease phenotypes in pediatric CD in Korea have been conducted^9,10,18^. In particular, there was only one study that evaluated the entire GI tract according to the Paris classification^10^. This is the first study in Korea to clearly compare the disease phenotypes of pediatric CD patients in Korea with those of a European cohort and investigate the impact of upper GI tract involvement on the disease course.

This study revealed that Korean pediatric patients had the distinct features of male predominance, less family history of IBD, more perianal disease, less isolated colonic involvement, and more upper GI tract involvement (74.4%) compared to those in the EUROKIDS registry^13^, consistent with other known studies^19,20^. As far as we know, this is the first study in Korea to assess disease phenotypes, investigate the factors associated with upper GI tract involvement, and compare the long-term disease outcomes according to upper GI tract involvement in pediatric CD patients.

Pediatric CD involving the upper GI tract has become increasingly recognized by the introduction of routine EGD and biopsies for all patients being evaluated for CD^11,17,21^. It is generally known that upper GI involvement is more prevalent in pediatric CD patients than in adult CD patients^22^. The frequency of upper GI tract involvement has been reported to vary widely^23,24^ and the definition of upper GI tract involvement in CD is still controversial. Some suggest that a wide spectrum of macroscopic and microscopic lesions should be considered proximal CD. Others recommend stricter classifications in terms of the mucosal alterations compatible with proximal CD. Our study chose the strict definition of upper GI tract involvement in CD, which was the same inclusion criterion as the EUROKIDS registry, and Korean patients showed significantly higher proportions of upper GI tract involvement compared to those in the EUROKIDS study (74.4% vs. 46.2%, *p* < 0.001). One possible explanation for this result is that the genetic differences between ethnicities may play a role in the expression of different phenotypes in pediatric CD. The other explanation is differences in the modalities of small bowel evaluation that were included in the upper GI tract evaluations between the two studies. Since this study was conducted between 2004 and 2019, more accurate modalities for small bowel evaluation (i.e., MRE or capsule endoscopy) in almost all the patients were used. However, since the EUROKIDS study was conducted between 2004 to 2009, less than half of the patients received advanced modalities for small bowel evaluation.

The comparison of the groups with and without upper GI tract involvement confirmed that the patients with upper GI tract involvement had a younger median age at diagnosis (13.4 vs. 14.8; *p* = 0.0133), as already suggested in the literature^25^. In addition, in the multivariate logistic regression model in this study, younger age at diagnosis (16 years or less, OR 2.594, *p* = 0.0139) was associated with more upper GI tract involvement, consistent with other published studies^13,26–28^. Moreover, previous studies reported that patients with upper GI tract involvement had more extensive disease at diagnosis compared to patients without such involvement, supporting our results that ileal involvement was associated with upper GI tract involvement (OR 2.293, *p* = 0.0176)^26,29–31^. However, compared to the EUROKIDS registry, the proportion of patients with upper GI involvement was higher in this study despite the older age at diagnosis, which can be explained by ethnic differences.

In this study, the disease course of pediatric patients with upper GI tract involvement at CD diagnosis did not appear to be significantly different from that of patients with non-upper GI tract involvement, inconsistent with the findings of previous studies. Also, when analysing disease outcomes according to jejunal involvement, there was no significant difference between the two groups, which is also different from previous reports^30^. Upper GI tract involvement was associated with poor prognosis such as prolonged hospitalization, increased need for surgery, and relapse in CD patients^26,31,32^. There are two possible explanations for our results. First, the subjects in this study had received more aggressive top-down scheduled anti-TNFα therapy without steroids, which resulted in favourable outcomes in patients with upper GI tract involvement. Anti-TNFα therapy was a factor significantly associated with upper GI tract involvement in this study (OR 2.292, *p* = 0.0365) (Table 4) and this was consistent with one study suggesting that patients with upper GI tract involvement required more aggressive therapeutic approaches^33^. Second, the increased detection of patients with upper GI tract involvement at the time of diagnosis, due to the increased use of EGD and small bowel imaging over time, may have resulted in the overdiagnosis of such involvement, considering even minor and particularly asymptomatic involvement as significant.

The strengths of this study are strict and uniform diagnostic criteria and classification of CD, standardized enrolment, and conduct at a single-center, meaning that all subjects received unified treatment strategies, minimizing the drawbacks of retrospective data analysis. Also, we analysed a relatively large number of patients (n = 312) and long-term outcomes (median follow-up of 6.6 years) compared to other pediatric studies. The limitations of our study are its retrospective nature and, consequently, unstructured patient follow-up. In addition, selection bias may have been introduced by excluding patients who had not undergone a complete GI evaluation.

In conclusion, in our study, 74.4% of the pediatric CD patients had upper GI tract involvement. A young age of ≤ 16 years and ileal involvement were identified as factors associated with upper GI tract involvement at the time of CD diagnosis. The long-term disease course did not appear different between the patients with and without upper GI tract involvement. However, patients with upper GI disease might require more aggressive therapeutic approaches.

## Data Availability

Please contact the corresponding author for data requests.
